# Bryostatin-1 vs. TPPB: Dose-Dependent APP Processing and PKC-α, -δ, and -ε Isoform Activation in SH-SY5Y Neuronal Cells

**DOI:** 10.1007/s12031-012-9816-3

**Published:** 2012-06-15

**Authors:** P. Yi, L. Schrott, T. P. Castor, J. S. Alexander

**Affiliations:** 1 Department of Molecular and Cellular Physiology, LSU Health, 1501 Kings Hwy, Shreveport, LA 71130 USA; 2 Department of Pharmacology, Toxicology and Neuroscience, LSU Health, 1501 Kings Hwy, Shreveport, LA 71130 USA; 3 Aphios Corporation, 3-E Gill St, Woburn, MA USA

**Keywords:** Alzheimer’s disease, PKC, sAPPα, APP, Alpha-secretase, Amyloid

## Abstract

Activation of the α-secretase processing pathway of amyloid precursor protein (APP) is recognized as an important mechanism which diverts APP processing from production of beta-amyloid (Aβ) to non toxic sAPPα, decreasing Alzheimer’s disease (AD) plaque formation and AD-associated cognitive deficits. Two potent classes of PKC modulators can activate the α-secretase pathway, the benzo/indolactams and bryostatin/bryologues. While both modulate PKC-dependent APP processing, no direct comparisons of their relative pharmacological potencies have been accomplished which could assist in the development of AD therapies. In this study, we measured the activation of α-secretase APP processing and PKC-α, -δ, and -ε induced by the benzolactam-APP modulator TPPB and bryostatin-1 in the neuroblastoma cell line SH-SY5Y which expresses APP and α- and β-secretase processing mechanisms. Bryostatin-1 produced a more rapid, potent, and sustained activation of α-secretase APP processing than TPPB and selectively activated PKC-δ and PKC-ε. Although TPPB also activated α-secretase, its potency was approximately 10- to 100-fold lower, possibly reflecting lower PKC-δ and -ε activation. Because bryostatin-1 is a highly potent PKC-δ and -ε activator which activates α-secretase APP processing, further characterization of bryostatin-1/bryologues may help refine their use as important tools for the clinical management of AD.

## Introduction

Despite more than a century of intensive research on Alzheimer’s disease (AD), few effective treatment options have been developed (Galimberti and Scarpini 2011). Currently, AD is treated using cholinesterase inhibitors (donepezil, galantamine, rivastigmine, tacrine) which support cholinergic signaling, and memantine, an *N*-methyl-d-aspartate receptor antagonist, which reduces glutamate-dependent excitotoxicity, Ca^2+^ influx, and oxidant stress in neurons (Tayeb et al. 2012). Both drug classes provide only marginal benefit, and more effective therapies are still required to treat AD. AD progression is associated with the appearance of extracellular “plaques” which contain β-amyloid (Aβ), as well as neurofibrillary tangles consisting of hyper-phosphorylated tau protein. Mechanistically, β-amyloid plaques are thought to provoke progressive neurodegeneration and behavioral and cognitive disturbances in AD.

Several other drug classes, e.g., non-steroidal anti-inflammatory drugs (NSAIDs) and “statins,” inhibitors of HMG-coA reductase, may also reduce formation and persistence of Aβ and have been suggested as potential adjuvant therapies for AD (Fonseca et al. 2010). Mechanistically, both statins and NSAIDs inhibit Rho G, Rho-kinase (ROCK), and Rac-1 activity, implying that these pathways may contribute to Aβ accumulation, AD plaques, and dementia, at least in mouse models of AD (Kurata et al. 2011). While intriguing, the introduction of statins and NSAIDs as adjuvant therapies in AD still requires clinical validation but may provide important clues for AD therapy (Hoyer and Reiderer 2007). Additionally, several phytochemicals (cannabinoids, physostigmine, and nicotine) and dietary modulators (curcumin, resveratrol, gingko, saffron, lemon balm, sage) have also been suggested as possible treatments for AD based on their effects on cholinergic signaling, oxidant formation, and processing of amyloid precursor protein (APP) (Nitsch et al. 1992; Howes and Perry 2011). The proteolytic processing of APP into extracellular Aβ deposits involves the action of β- and γ-secretases. Based on this understanding of APP processing, inhibitors of both β- and γ-secretases have been evaluated as Aβ plaque-suppressing therapeutics in AD.

Inhibitors of β-secretase (BACE-1) include diphenylurea, derivatives of hydroxyethylamine, and celastol, all of which at least partly block conversion of APP to Aβ. Τhese drugs have been extensively studied in AD therapy (Huang et al. 2010; Paris et al. 2010; Truong et al. 2010). While effective against β-secretase in vitro (Hills and Vacca 2007), these inhibitors have been less effective in in vivo/clinical AD studies. Similarly, γ-secretase modulators (“GSM”) have been proposed to reduce Aβ accumulation (Imbimbo and Giardina 2011). Several candidate GSMs, “DAPM”, and “DAPT” (Czvitkovich et al. 2011), NSAIDS (Ibuprofen, flurbiprofen), and purine analogs (Rivkin et al. 2010) were proposed as γ-secretase modulators, but clinical trials have been unsuccessful and a recent clinical trial with semagacestat (LY-450139) was halted because of potentially accelerated dementia.

While β- and γ-secretases cooperatively convert APP into Aβ, α-secretase is a competing pathway for APP which diverts the substrate APP into production of sAPPα, which is non toxic and effectively prevents the formation of amyloid plaques and AD-related dementia. α-secretase is actually a family of several proteins including tumor necrosis factor-α converting enzyme (“TACE”), a disintegrin and metalloprotease (‘ADAM’)-17 (Black et al. 2003), and ADAM-9, -10, and -19 (Asai et al. 2003; Tanabe et al. 2007). While many strategies are aimed to block disease progression in animal models of AD, activation of α-secretase processing of APP remains highly attractive because it competes with the β- and γ-secretase pathways for APP. Several cell signaling modules can activate α-secretase processing of APP, including Mints/X11s and Nogo-66 receptor (which bind APP), Rho GTPases, reticulon (RTN)-3 and -4, and the prolyl isomerase “Pin1.” These mechanisms suppress the formation of Aβ both in vitro and in vivo (Tang and Liou 2007) but have not yet been exploited therapeutically (Ikin et al. 2007).

One of the most potent and effective means of triggering α-secretase is by protein kinase C (PKC) activation. Several drugs which modulate PKC isoenzyme activity, particularly the PKC-δ and PKC-ε isoforms, potently activate α-secretase processing of APP, generating the non toxic sAPPα and preventing Aβ formation. Currently, PKC activators may represent the most important, novel, and specific treatments for AD. At least two important classes of agents can activate PKC-dependent APP processing at relatively low (μM to nM) doses: (1) the benzolactam/indolactams and (2) bryostatin-1 and related bryologues.

TPPB is a novel cell-permeable benzolactam-derived PKC activator (*K*_i_ = 11.9 nM for inhibition of phorbol 12,13 dibutryrate binding to PKC-α) which enhances sAPPα secretion in fibroblasts and PC12 neuronal cells (Ibarreta et al. 1999). The bryostatins are a family of ~20 natural marine cyclic macrolides (Pettit et al. 1982) with potent anti-cancer properties. Bryostatin-1 is a powerful PKC activator, potently stimulating PKC-δ and -ε isozymes at sub-nanomolar concentrations (Szallasi et al. 1994). Emerging evidence suggests that bryostatin-1 can enhance memory and cognition and helps stabilize or even reverse AD-associated dementia (Sun and Alkon 2010). Although bryostatin-1 and TPPB share similar targets, pharmacology, and applications in AD research, a direct comparison of benzolactam and bryostatin-1 as APP modulators has not yet been carried out in an in vitro neuron model, which could provide important insights on their use in mechanism-based AD therapy. This current study evaluates dose- and time-dependent activation of α-secretase (sAPPα secretion) and PKC-α, -δ, and -ε isoform activation in the SH-SY5Y cell line in response to bryostatin-1 and TPPB.

## Materials and Methods

### Test Agents

Bryostatin-1 in ethanol stock solutions was provided by Aphios Corporation (Woburn, MA, USA). The benzolactam APP modulator TPPB [(2*S*,5*S*)-(*E*,*E*)-8-(5-(4-(trifluoromethyl) phenyl)-2,4-pentadienoylamino) benzolactam] (CAS#497259-23-1) was purchased from Reagent 4 Research LLC (Hangzhou, China) and was diluted in ethanol. All ethanol concentrations in this study were below 0.1 % in cellular assays.

### Cells and Cell Culture

SH-SY5Y neuroblastoma cells (Kohl et al. 1980) were selected for studying neuronal APP metabolism because they retain a neuronal phenotype, possess a full complement of APP-processing and signaling pathways, and have been well characterized in AD studies (Canet-Aviles et al. 2002). SH-SY5Y neuroblastoma cells were obtained from American Type Culture Collection (Manassas, VA, USA) and were maintained in DMEM (4.5 g/L glucose), 10 % fetal bovine serum (FBS), and 1 % antibiotic/antimycotic. SH-SY5Y cells were subcultured into six-well polystyrene tissue culture dishes and grown to confluency prior to use in APP processing and PKC activation studies.

### Treatment Protocols

Cells were grown to confluency in 10 % serum-supplemented medium. Prior to experiments, culture medium was replaced with serum and protein-free (0 %) medium for 2 h before drug treatments to remove APP/sAPPα present in FBS and to permit filter concentration of sAPPα. Dilutions of bryostatin-1 or TPPB were added to serum-free culture medium at various concentrations and treatments maintained for 3 h, except in time course experiments where several time points up to 24 h were taken. Pilot studies showed no change in SH-SY5Y appearance, attachment, or cell density for at least 24 h under these conditions.

### Measurements of α-Secretase Activity (Production of sAPPα)

Activation of α-secretase was monitored by measuring the secretion of the soluble 93-kDA cleavage product of APP (“sAPPα”) generated by α-secretase. After drug treatments, serum-free culture medium was harvested and centrifuged at 14,000 × *g* for 15 min across a 10-kDA cutoff Amicon membrane ultra-centrifugal filter (Millipore Corporation, Billerica, MA, USA). Concentrated supernatants containing sAPPα were resuspended in 50 μL of Laemmli sample buffer, heated (100 °C, 5 min), electrophoresed on 10 % SDS/PAGE gels, and immunoblotted to 0.2 μm nitrocellulose membranes to quantify sAPPα. Membranes were blocked with 5 % nonfat dry milk/PBS for 1 h to prevent nonspecific antibody binding. Blocked membranes were incubated overnight at 4 °C with 1:500 diluted 6E10 monoclonal antibody in 0.1 % milk powder (Covance, Dedham, MA, USA). 6E10 antibody (Covance, Berkeley, CA, USA) is raised against the sequence DAEFRHDSGYEVHHQK which is common to APP, β-amyloid, and sAPPα. sAPPα corresponds to 90 kDA 6E10 antibody reactive band recovered from the medium-soluble fraction (Yang et al. 2007). After washing, membranes were incubated for 2 h at 25 °C with horseradish peroxidase (HRP)-conjugated anti-mouse IgG secondary antibody (diluted 1:5,000, Sigma). HRP-bound signal was detected using enhanced chemiluminescence (“ECL plus,” GE Healthcare, UK) to expose X-ray film (SRX101 film, KONICA Corp.). Band intensities were quantified by densitometry analysis using an HP Scanjet 3970 Densitometer and Image-J image analysis system software (Ver. 3.0, NIH). Control band densities on each blot (sAPPα, PKC-α, -d, or -ε) were set as 100 %, and changes in recovery of target proteins in response to drug treatments were expressed as the normalized percent of each control. All experiments were performed in triplicate (*n* = 3).

### Activation of PKC Isoforms by TPPB and Bryostatin-1

Measurements of cytosolic and membrane-associated levels of PKC-α, -δ, and -ε isoenzymes were used to assess PKC translocation in response to bryostatin-1 or TPPB as described by Racchi et al. (1994) with modifications (Ibarreta et al. 1999). Briefly, following treatments, cells were washed twice with ice-cold PBS, scraped using a rubber policeman, and collected by low-speed centrifugation at 1,500 × *g* rpm for 5 min. These pellets were resuspended and vortexed in “homogenization buffer” (50 mM Tris HCl [pH 7.4], 150 mM NaCl, 1 mM EDTA, and mixed protease inhibitors (Sigma)). Cells were centrifuged at 15,000 × *g* for 30 min. These supernatants were reserved as “cytosol” fractions. Pellets from this spin were homogenized in this same buffer containing 1.0 % Triton X-100/1.0 % NP-40, sonicated, incubated on ice (45 min), and centrifuged again at 15,000 × *g* for 30 min. The supernatants from this batch were “membrane” fractions. After protein determination (BCA method, Biorad), protein samples were diluted in 2× Laemmli sample buffer, heated (100 °C, 5 min), electrophoresed on 10 % SDS/PAGE gels, and immunoblotted to 0.2 μm nitrocellulose for probing with 1:500 diluted anti-PKC-α [antibody h-7, sc-8393], -δ [antibody G-9, sc-8402], and -ε [antibody E-5, sc-1681] antibodies (Santa Cruz Biotechnology, Santa Cruz, CA, USA). H-7 antibody is a mouse monoclonal antibody raised against amino acids 645–672 on human PKC-α which recognizes a single 80-kDA PKC-α isoform. G-9 antibody is a mouse monoclonal antibody raised against amino acids 647–673 of rat PKC-δ which recognizes a single 78-kDA band, and E-5 antibody is a mouse monoclonal antibody which was raised against amino acids 705–737 of human PKC-ε and recognizes a single 90-kDA PKC-ε band. PKC isoform bands were visualized using HRP-conjugated 2° antibody (1:5,000) with ECL plus (GE Healthcare, UK) with SRX101 film. Band intensities were quantified by densitometric analysis using an HP Scanjet 3970 Densitometer and Image-J image analysis system software (Ver. 3.0, NIH).

### Statistical Analysis

All data are presented as mean ± standard deviation and were analyzed by one-way ANOVA with Dunnett’s post-testing (Graphpad Instat 3 Software, San Diego, CA, USA). A value of *p* < 0.05 was considered as statistically significant.

## Results

### Induction of sAPPα Secretion by Bryostatin-1 and TPPB in SH-SY5Y Neuroblastoma Cells

**Fig. 1:**
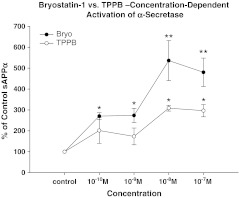
Increase in α-secretase activity induced by different concentrations of TPPB and bryostatin-1 in SH-SY5Y neuroblastoma cells for 3 h. Bryostatin-1 significantly induced sAPPα release at 10^−7^ M (**p < 0.01), 10^−8^ M (**p < 0.01), 10^−9^ M (*p < 0.05), and 10^−10^ M (*p < 0.05). TPPB also induced sAPPα release, but to a lesser extent, and only at 10^−7^ M (*p < 0.05) and 10^−8^ M (*p < 0.05)

sAPPα secretion induced by treatment of SH-SY5Y neuroblastoma cells with different concentrations of bryostatin-1 or the benzolactam modulator TPPB was initially studied at 3 h. Bryostatin-1 significantly induced sAPPα release at 10^−7^ M (**p < 0.01), 10^−8^ M (**p < 0.01), 10^−9^ M (*p < 0.05), and 10^−10^ M (*p < 0.05). TPPB also induced sAPPα release, but to a lesser extent and only at 10^−7^ M (*p* < 0.05) and 10^−8^ M (*p* < 0.05) (Fig. 1).

**Fig. 2:**
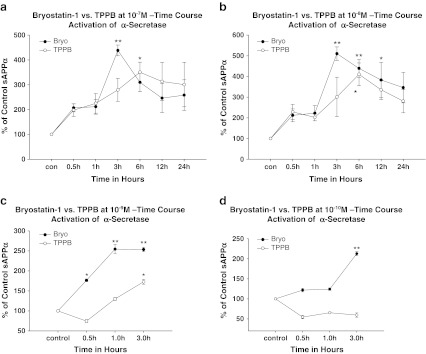
**a** Time course of α-secretase activity induced by bryostatin-1 and TPPB at 10^−7^ M in SH-SY5Y neuroblastoma cells. Bryostatin-1 induced a significant release of sAPPα at 3 h (**p < 0.01), and TPPB induced the release of sAPPα at 6 h (*p < 0.05). **b** Time course of α-secretase activity induced by bryostatin-1 and TPPB at 10^−8^ M in SH-SY5Y neuroblastoma cells. Bryostatin-1 induced a significant release of sAPPα at 3 h (**p < 0.01), 6 h (**p < 0.01), and 12 h (*p < 0.05); TPPB induced the release of sAPPα at 6 h (*p < 0.05). **c** Time course of α-secretase activity induced by bryostatin-1 and TPPB at 10^−9^ M in SH-SY5Y neuroblastoma cells. Bryostatin-1 induced a significant release of sAPPα beginning at 0.5 h (*p < 0.05), 1 h (**p < 0.01), and 3 h (**p < 0.01); TPPB induced the release of sAPPα at 3 h (*p < 0.05). **d** Time course of α-secretase activity induced by bryostatin-1 and TPPB at 10^−10^ M in SH-SY5Y neuroblastoma cells. Bryostatin-1 induced a significant release of sAPPα at 3 h (**p < 0.01); 10^−10^ M TPPB did not significantly induce sAPPα release

A time course study in SH-SY5Y neuroblastoma cells induced by bryostatin-1 and TPPB showed that, at 10^−7^ M, bryostatin-1 significantly increased the release of sAPPα at 3 h (**p < 0.01); TPPB was able to significantly increase sAPPα at 6 h (*p < 0.05) (Fig. 2a). At 10^−8^ M, bryostatin-1 significantly induced sAPPα release at 3 h (**p < 0.01), 6 h (**p < 0.01), and 12 h (*p < 0.05). At this dose, TPPB only significantly increased the release of sAPPα at 6 h (*p < 0.05) (Fig. 2b). Bryostatin-1 at 10^−9^ M induced the significant release of sAPPα at 0.5 h (*p < 0.05), which persisted at 1 h (**p < 0.01) and 3 h time points (**p < 0.01). By comparison, TPPB only induced the significant release of sAPPα at 3 h (*p < 0.05) (Fig. 2c). Bryostatin-1 at 10^−10^ M induced the significant release of sAPPα at 3 h (**p < 0.01); however, TPPB at 10^−10^ M did not significantly increase the release of sAPPα at any of the time points tested (Fig. 2d).

### Activation of PKC Isoforms by Bryostatin-1 or TPPB in SH-SY5Y Neuroblastoma Cells

**Fig. 3:**
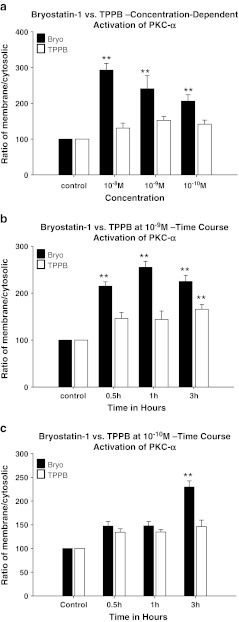
**a** PKC-α translocation induced by different concentrations of bryostatin-1 and TPPB in SH-SY5Y neuroblastoma cells at 3 h. Bryostatin-1 significantly induced the translocation of PKC-α at 10^−8^ M (**p < 0.01), 10^−9^ M (*p < 0.05), and 10^−10^ M (*p < 0.05); TPPB did not induce a significant PKC-α translocation. **b** Time course of PKC-α translocation induced by 10^−9^ M bryostatin-1 or TPPB in SH-SY5Y neuroblastoma cells at 0.5, 1, and 3 h. A total of 10^−9^ M bryostatin-1 significantly induced the translocation of PKC-α at 0.5 h (**p < 0.01), 1 h (*p < 0.05), and 3 h (**p < 0.01); 10^−9^ M TPPB only induced a significant PKC-α translocation at 3 h. **c** Time course of PKC-α translocation induced by 10^−10^ M bryostatin-1 and TPPB in SH-SY5Y neuroblastoma cells. A total of 10^−10^ M bryostatin-1 significantly induced the translocation of PKC-α at 3 h (**p < 0.01); 10^−10^ M TPPB did not significantly induce PKC-α translocation at any of the time points tested

**Fig. 4:**
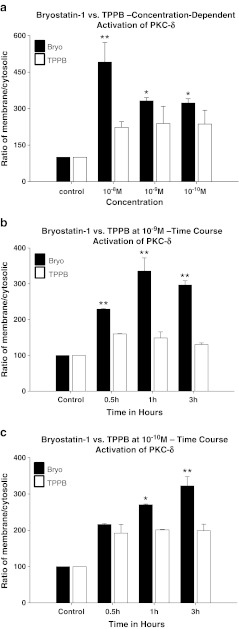
**a** PKCδ translocation induced by different concentrations of bryostatin-1 and TPPB in SH-SY5Y neuroblastoma cells for 3 h. Bryostatin-1 significantly induced the translocation of PKCδ at 10^−8^ M (**p < 0.01), 10^−9^ M (*p < 0.05), and 10^−10^ M (*p < 0.05), but TPPB did not significantly induce PKCδ translocation at any concentration tested. **b** Time course of PKC-δ translocation induced by bryostatin-1 and TPPB at 10^−9^ M in SH-SY5Y neuroblastoma cells. Bryostatin-1 induced a significant PKC-δ translocation beginning at 0.5 h (**p < 0.01), 1 h (**p < 0.01), and 3 h (**p < 0.01); 10^−9^ M TPPB did not significantly induce PKC-δ translocation at any of the times tested. **c** Time course of PKC-δ translocation induced by bryostatin-1 and TPPB at 10^−10^ M in SH-SY5Y neuroblastoma cells. Bryostatin-1 induced a significant PKC-δ translocation beginning at 1 h (*p < 0.05) and 3 h (**p < 0.01); 10^−10^ M TPPB did not significantly induce PKC-δ translocation at any of the times tested

**Fig. 5:**
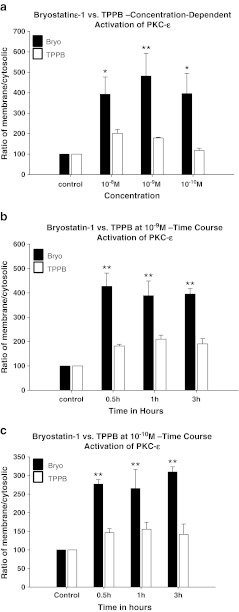
**a** PKC-ε translocation induced by different concentrations of bryostatin-1 and TPPB in SH-SY5Y neuroblastoma cells at 3 h. Bryostatin-1 significantly induced the translocation of PKC-ε at 10^−8^ M (*p < 0.05), 10^−9^ M (**p < 0.01), and 10^−10^ M (*p < 0.05); TPPB did not significantly induce PKC-ε translocation at any of the concentrations tested. **b** Time course of PKC-ε translocation induced by bryostatin-1 and TPPB at 10^−9^ M in SH-SY5Y neuroblastoma cells. Bryostatin-1 induced a significant PKC-ε translocation beginning at 0.5 h (**p < 0.01), 1 h (**p < 0.01), and 3 h (**p < 0.01); TPPB did not significantly induce PKC-ε translocation at any of the time points tested. **c** Time course of PKC-ε translocation induced by bryostatin-1 and TPPB at 10^−10^ M in SH-SY5Y neuroblastoma cells. Bryostatin-1 induced a significant PKC-ε translocation beginning at 0.5 h (**p < 0.01), 1 h (**p < 0.01), and 3 h (**p < 0.01); 10^−10^ M TPPB did not significantly induce PKC-ε translocation at any of the time points tested

PKC activation was measured by comparing the cytosol to membrane translocation of PKC isoforms α, δ, and ε (relative to actin) following treatment with bryostatin-1 or TPPB. Confluent cultures of SH-SY5Y neuroblastoma cells were treated with different concentrations of either bryostatin-1 or TPPB for time points up to 3 h, when membrane and cytosolic pools of PKCs were isolated by differential detergent extraction, and Western-blotted for each individual isoform. Actin was also Western-blotted in each fraction to ensure equal loading. At 3 h, 10^−8^, 10^−9^, and 10^−10^ M bryostatin-1 significantly increased the activation of PKC-α in SH-SY5Y cells (**p < 0.01) (Fig. 3a); 10^−8^, 10^−9^, and 10^−10^ M TPPB did not activate PKC-α (Fig. 3a). A time course activation study of 10^−9^ M bryostatin and 10^−9^ M TPPB showed that 10^−9^ M bryostatin-1 activated PKC-α at 0.5, 1, and 3 h (**p < 0.01) (Fig. 3b); 10^−9^ M TPPB only significantly activated PKC-α at 3 h (**p < 0.01) (Fig. 3b). Bryostatin-1 at 10^−10^ M activated PKC-α at 3 h (**p < 0.01); 10^−10^ M TPPB did not activate PKC-α at any time point tested. A dose-dependent comparison of bryostatin-1 and TPPB showed that, at 3 h, PKC-δ was activated by 10^−8^ M bryostatin-1 (**p < 0.01), 10^−9^ M bryostatin-1 (*p < 0.05), and 10^−10^ M bryostatin-1 (*p < 0.05) (Fig. 4a); TPPB did not activate PKC-δ at any concentration tested at 3 h (Fig. 4a). A time course study of 10^−9^ M bryostatin-1 and TPPB showed that 10^−9^ M bryostatin-1 significantly activated PKC-δ at 0.5 h (**p < 0.01), 1 h (**p < 0.01), and 3 h (**p < 0.01); by comparison, 10^−9^ M TPPB did not activate PKC-δ at any time point tested (Fig. 4b). A time course study of 10^−10^ M bryostatin-1 and TPPB showed that 10^−10^ M bryostatin-1 significantly activated PKC-δ at 1 h (*p < 0.05) and 3 h (**p < 0.01); by comparison, 10^−10^ M TPPB did not activate PKC-δ at any time point (Fig. 4c). A dose-dependent comparison of bryostatin-1 and TPPB showed that, at 3 h, PKC-ε was activated by 10^−8^ M bryostatin-1 (*p < 0.05), 10^−9^ M bryostatin-1 (**p < 0.01), and 10^−10^ M bryostatin-1 (*p < 0.05) (Fig. 5a); TPPB did not activate PKC-ε at any concentration tested at 3 h (Fig. 5a). A time course study of 10^−9^ M bryostatin-1 and TPPB showed that 10^−9^ M bryostatin-1 significantly activated PKC-ε at 0.5 h (**p < 0.01), 1 h (**p < 0.01), and 3 h (**p < 0.01) (Fig. 5b); by comparison, 10^−9^ M TPPB did not activate PKC-ε at any of the time points tested (Fig. 5b). A time course study of 10^−10^ M bryostatin-1 and TPPB showed that 10^−10^ M bryostatin-1 significantly activated PKC-ε at 0.5 h (*p < 0.01), 1 h (*p < 0.01), and 3 h (**p < 0.01) (Fig. 5c); by comparison, 10^−10^ M TPPB did not activate PKC-δ at any of the time points tested (Fig. 5c).

## Discussion

The presence of Aβ-laden cerebral plaques in AD, a central feature of Alzheimer’s disease (AD), is thought to contribute to the disturbances in nerve structure, function, and memory which characterize AD dementia (Almeida et al. 2005; Hsieh et al. 2006; Selkoe 2002). Aβ plaques are neurotoxic and thought to provoke detrimental effects on memory, cognitive function (Ono and Yamada 2011; Zhang et al. 2011), and synaptic organization (Cleary et al. 2005; Shankar et al. 2008). Paradoxically, normal synaptic activity generates Aβ (Cirrito et al. 2005; Kamenetz et al. 2003) and very low (~pM) levels of Aβ enhance synaptic plasticity (Puzzo et al. 2008). Therefore, while excessive Aβ produces pathology in AD, very limited APP processing to Aβ may normally participate in synaptic function (albeit at lower levels than those seen in AD). Because the disproportionate formation of Aβ can be prevented by activation of the α-secretase pathway, agents which activate this pathway may have important future applications in AD treatment.

There has recently been an intense focus on research governing the molecular events controlling α-secretase processing of APP as a powerful prophylactic and therapeutic approach for AD. α-Secretase processing can be activated by cholinergic g-protein-coupled receptors (Nitsch et al. 1992), receptor tyrosine kinases, MAP kinases, PI3-kinase, cAMP and calcium kinases, and several PKC isoforms (Postina 2012). In particular, activators of the “novel” PKC isoforms (PKC-δ and PKC-ε) potently activate α-secretase processing of APP and may represent novel and specific targets for AD therapy. PKC isoforms exhibit diverse target specificities reflecting heterogeneous expression in different cell and tissue types, cell compartment, and duration and mode of activation. Since PKC activators have emerged as highly potently α-secretase activators, there has been an intensive investigation to identify potent and selective PKC-α-secretase activators with low toxicity and carcinogenicity and high penetration of the blood–brain barriers.

Several AD research groups have investigated PKC activation as a central regulator of APP processing, AD pathophysiology, and cognitive impairment (Ruiz-Leon and Pascual 2001). In AD, brain PKC levels are decreased, which may partly explain some of the defects in APP processing that characterize AD (Govoni et al. 1993). In AD, PKC-β protein levels are reduced, but PKC-α, -β , and − γ mRNAs were unaffected, consistent with rapid PKC-β proteolysis (Chachin et al. 1996). AD dementia has also been associated with lower levels of PKC activation (Wang et al. 1994). Aβ and APP reduce intra-cerebrellar PKC-ε levels (de Barry et al. 2010; Hongpaisan et al. 2011; Liron et al. 2007), PKC phosphorylation (Moriguchi et al. 2006), and PKC activity (Lee et al. 2004), suggesting a positive feedback loop of Aβ formation and PKC suppression. Age-related decreases in PKC may also lead to defects in APP processing and contribute to AD pathology (Pascale et al. 1998). These findings all support decreased PKC and PKC hypofunctioning as underlying causes of AD progression. Consistent with these findings, activation of PKCs (especially novel isoforms) and α-secretase has been shown to ameliorate pathophysiology and cognitive impairment in AD (Ruiz-Leon and Pascual 2001).

PKC isoforms -α, -δ, and -ε potently regulate APP processing (Yeon et al. 2001), and differential activation of these isoforms has important implications for the design of mechanism-based therapeutics in AD. PKC-α is a “classical” PKC requiring Ca^2+^, diacylglycerol, and phospholipid for activation (Konopatskaya and Poole 2010). PKC-δ is a “novel” PKC isoform which modulates learning and memory (Sun and Alkon 2010), which is activated by *cis*-unsaturated fatty acids (Khan et al. 1993) and by 3,4 diphosphatidyl- and 1,3,4 triphosphatidyl-inositol (PdIns) (Toker et al. 1994). PKC-δ activation by PdIns species may provide important links between phospholipase C signaling and PKC-δ activation. PKC-δ was originally cloned from brain tissue (Ono et al. 1987) and is a prominent albeit heterogeneously distributed PKC isoform in the post-natal brain (Chen and Hillman 1993; Ogita et al. 1992). PKC-δ (but not PKC-α or PKC-ε) also activates MEK-ERK signaling (Ueda et al. 1996).

PKC-δ plays a specific role in stimulating neuritogenesis in PC12 cells (O'Driscoll et al. 1995). PKC-ε (Koide et al. 1992), another “novel” PKC is expressed at high levels in brain (Mischak et al. 1993; Van Kolen et al. 2008; Wetsel et al. 1992) and has been cited as an important PKC regulating Aβ and sAPPα ratios (Zhu et al. 2001). PKC-ε activators, like bryostatin-1, directly enhance cognition (Wang et al. 2008) and limit dementia in mouse models of AD (Hongpaisan et al. 2011) through several mechanisms which may include (1) Ca^2+^ flux, (2) GABA(A)_R_-regulated post-synaptic currents, and (3) cholinergic signaling (Van Kolen et al. 2008).

Acute treatment with tetradecanoyl phorbol acetate (TPA) activates α-secretase via PKC-α (Racchi et al. 2003). Neurons treated with TPA for 24 h down regulated PKC-α, -β, and -γ but maintained PKC-δ and -ε (Burry 1998). Chronic TPA treatment down regulated PKC-α and increased PKC-ε expression (Da Cruz e Silva et al. 2009). Importantly, chronic TPA treatment decreases sAPPα and increases Aβ formation. Therefore, acute TPA exposure enhances sAPPα and decreases Aβ; chronic TPA treatment increases Aβ (Da Cruz e Silva et al. 2009).

PKC-dependent APP modulators remain highly attractive strategies for AD therapy because of their high potency, specificity, low potential for carcinogenesis, and high anti-inflammatory potential (Carpenter and Alexander 2008). Two important members of this class include bryostatin-1 and TPPB. Bryostatin-1 is a cyclic macrolide lactone modulator of PKC that occurs at low concentrations (5–25 ppm) in the marine bryozoan, *Bugula neritina*, an invasive type of “sea moss” found in coastal California and Hawaii. Bryostatin-1, a highly potent PKC activator, enhances cognition and memory consolidation independently of its ability to stabilize AD-related dementia (Sun and Alkon 2010). Bryostatin-1 is effective as a chronic activator of APP processing, even in cells with AD defects, because it does not decrease PKC-δ or -ε like phorbol esters and does not lead to PKC isoform suppression. For example, fibroblasts from patients with AD had defects in APP secretion following phorbol ester stimulation (Bergamaschi et al. 1995), while sub-nanomolar concentrations of Bryostatin-1 dramatically enhanced the generation of non-amyloidogenic sAPPα (Nelson et al. 2009). Bryostatin-1 reduces amyloid plaques (detected as Aß40 and Aß42 in AD) in the brains of transgenic AD mice and improved behavioral outcomes and the rate of death in AD transgenic mice. Bryostatin-1 also enhances cognition and memory consolidation outside the settings of AD-dementia models (Sun and Alkon 2010). Since bryostatin-1 produces neuroprotection in AD animal models and independently enhances cognition and memory, it is possible that bryostatin-1 treatment may be able to restore as well as prevent AD-associated dementia.

By comparison, TPPB is a novel, synthetic cell-permeable benzolactam PKC activator which has been used as a standard APP modulator (Kozikowski et al. 2003). TPPB binds “classical” (with a *K*_i_ of 11.9 nM for PKC-α) and “novel” PKC isoforms to stimulate sAPPα processing (Kozikowski et al. 2003; Yang et al. 2007). TPPB stimulates α-secretase in human fibroblasts (AG06848 cells) at concentrations as low as 100 nM (10^−7^ M) to enhance non-amyloidogenic processing of APP to sAPPα. Currently, the toxicological properties of TPPB are not fully known. An important goal in PKC–APP modulator drug design must be to overcome the potential carcinogenic properties of benzolactams/indolactams because TPPB, like TPA and other phorbol esters, may still retain tumor-promoting activity (Kozikowski et al. 2003). Therefore, although benzolactams like TPPB display reduced tumorigenic activity compared to phorbol esters (Irie et al. 2004; Nakagawa et al. 2001), this concern becomes increasingly more significant considering the relatively high doses (~10^−7^ M) of TPPB needed to reach equivalent α-secretase activation as bryostatin-1 which may hamper its use in human AD therapy. There are, of course, relative factors of availability and cost of bryostatin-1 versus reduced potency and higher toxicity of TPPB which can ultimately determine clinical use.

Our current study compared the pharmacokinetics of α-secretase APP processing and PKC-α, -δ, and -ε isoform activation by bryostatin-1 and TPPB in SH-SY5Y neuroblastoma cells. Bryostatin-1 significantly induced sAPPα release from 10^−7^ to 10^−10^ M. By comparison, TPPB induced sAPPα release at 10^−7^ and 10^−8^ M. Even at effective doses, the magnitude of TPPB activation of α-secretase was lower than bryostatin-1. Time course studies also show that 10^−7^ M bryostatin-1 significantly induced sAPPα release by 3 h. TPPB also induced sAPPα release at 10^−7^ M but required 6 h to significantly induce sAPPα release at this dose. Bryostatin-1 at 10^−8^ M significantly increased sAPPα release at 3, 6, and 12 h; 10^−8^ M TPPB only induced significant sAPPα release at 6 h. Bryostatin-1 at 10^−9^ M significantly increased sAPPα secretion beginning at 30 min through 3 h, while 10^−9^ M TPPB was only able to induce the release of sAPPα at 3 h. Lastly, at 10^−10^ M, bryostatin-1 significantly increased sAPPα release at 3 h, where 10^−10^ M TPPB did not significantly increase sAPPα release. Both time and dose comparisons indicate that bryostatin-1 induces sAPPα release in SH-SY5Y neuroblastoma cells at one to two orders of magnitude greater potency and at earlier time points than TPPB. Bryostatin-1 therefore represents a highly active APP modulator which could be used as a low-dose therapy for AD.

Novel PKC activators are important candidate treatments for AD because they activate beneficial APP processing through α-secretase. We compared how PKC-α (a “classical” PKC), -δ, and -ε (novel PKC isoforms) were activated in neuroblastoma cells by bryostatin-1 and TPPB as a function of time and concentration. Bryostatin-1 significantly induced PKC-α, -δ, and -ε activation at 10^−8^, 10^−9^, and 10^−10^ M in SH-SY5Y cells. TPPB, however, was only able to significantly activate PKC-α at 10^−9^ M after 3 h. Interestingly, this activation was not seen at higher doses or at earlier time points. Time course experiments showed that 10^−9^ M bryostatin-1 induced a significant and persistent activation of PKC-α, -δ, and -ε beginning at 30 min and which persisted for at least 3 h. At 10^−10^ M, bryostatin-1 also induced PKC-α activation at 3 h; 10^−10^ M bryostatin-1 triggered significant PKC-δ activation at 1 h, which persisted for 3 h. At 10^−10^ M, bryostatin-1 also triggered PKC-ε activation by 30 min, which persisted for 3 h. TPPB did not significantly induce PKC-δ or PKC-ε activation at any concentration. Although TPPB has been demonstrated to activate PKC in this cell line (Yang et al. 2007), this effect was seen with TPPB doses in the order of 10–1,000 × *g* greater than those used for bryostatin-1 in the current studies. Therefore, when comparing bryostatin-1 and TPPB, a recognized APP modulator, bryostatin-1 was found to potently activate PKC-ε translocation in SH-SY5Y cells at 10^−8^, 10^−9^, and 10^−10^ (at 3 h). Bryostatin-1 also appears to be a more active, rapid, and persistent APP modulator which reflects its greater specificity toward PKC-δ or PKC-ε isoform activation at low doses.

## Conclusions

Here, we found that bryostatin-1 exerted a more rapid, potent, and sustained activation of APP processing (in particular, the selective activation of the α-secretase amyloid processing pathway), which was associated with its more potent and specific activation of PKC-δ and PKC-ε at low doses. The further refinement of bryostatin-1 pharmacology in PKC mobilization and α-secretase activation may help move this drug class more rapidly into clinical treatment for AD.
